# Quasi-Static and High Strain-Rate Behavior of Carbon Fiber Reinforced Modified BOFS Concrete

**DOI:** 10.3390/ma18194497

**Published:** 2025-09-27

**Authors:** Yeou-Fong Li, Chun-Wei Chien, Jin-Yuan Syu, Chih-Hong Huang, Wen-Shyong Kuo, Ying-Kuan Tsai

**Affiliations:** 1 Department of Civil Engineering, National Taipei University of Technology, Taipei 10608, Taiwan; cg11cg17@gmail.com (C.-W.C.); t9679010@ntut.org.tw (J.-Y.S.); 2 Department of Architecture, National Taipei University of Technology, 1, Sec. 3, Chung-Hsiao E. Rd., Taipei 10608, Taiwan; 3 Department of Aerospace and Systems Engineering, Feng Chia University, Taichung 40724, Taiwan; wskuo@fcu.edu.tw; 4 Department of Civil Engineering, National Yang Ming Chiao Tung University, Hsinchu 30010, Taiwan; jeremytsai0406@nycu.edu.tw

**Keywords:** carbon fiber, modified basic oxygen furnace slag, fiber-reinforced concrete, thermogravimetric analysis, single-fiber tensile test, split Hopkinson pressure bar

## Abstract

This study examines the mechanical properties of concrete in which natural aggregates are entirely replaced by modified basic oxygen furnace slag (MBOFS) and reinforced with chopped carbon fibers, under both dynamic and quasi-static loading conditions. The carbon fiber (CF) was subjected to heat treatment and pneumatic dispersion prior to mixing, and its performance was validated using thermogravimetric analysis (TGA) and single-fiber tensile tests. The experimental program included tests on workability, compressive strength, flexural strength, splitting tensile strength, impact resistance, and high strain rate behavior using the reverse split Hopkinson pressure bar (RSHPB) method. Thermogravimetric analysis (TGA) and scanning electron microscope (SEM) confirmed that heat treatment removed surface sizing from carbon fibers (CF) with minimal effect on tensile strength. Replacing natural aggregates with MBOFS reduced slump but enhanced compressive, flexural, and splitting tensile strength. Incorporating 1% chopped CF further improved mechanical performance: 6 mm CF increased compressive strength, while 12 mm CF enhanced flexural and splitting tensile strength. Impact resistance improved with CF addition, with 12 mm CF slightly outperforming 6 mm. RSHPB tests showed higher dynamic strength for 6 mm CF specimens, with both strength and dynamic increase factor rising with strain rate and gas pressure.

## 1. Introduction

Natural sand and gravel are among the most widely consumed materials worldwide and are indispensable in civil engineering and related industries. However, the rapid growth of construction and manufacturing sectors has intensified the extraction of these natural aggregates, leading to significant environmental and ecological concerns [1,2,3]. The utilization of recycled materials as substitutes has therefore been considered a promising strategy to reduce dependence on natural resources.

The steel industry plays a critical role in national economic growth and remains a key driver of industrial development. Conventional steelmaking processes generate two major by-products: blast furnace slag and basic oxygen furnace slag (BOFS). Owing to its high hardness and wear resistance, the utilization of BOFS in line with the principles of the circular economy—widely promoted worldwide—has become a priority for steel producers [4,5,6]. BOFS also exhibits favorable properties, including high wear resistance, high hardness, and low leaching toxicity [7,8,9], which make it suitable for applications such as road base materials [10] and artificial reef structures supporting algal colonization [11]. Furthermore, when used as a substitute for natural aggregates in concrete, BOFS has been shown to significantly improve the compressive strength of the mixture [12].

During steelmaking, the addition of limestone and dolomite introduces free calcium oxide (f-CaO) and free magnesium oxide (f-MgO) into BOFS. These oxides undergo hydration, forming calcium hydroxide (Ca(OH)_2_) and magnesium hydroxide (Mg(OH)_2_). In addition, metallic iron present in the slag can oxidize when exposed to water and air. Such reactions may cause volume expansion in BOFS, thereby compromising the durability of concrete structures [13,14,15,16,17]. To mitigate these issues, various techniques have been developed to reduce the f-CaO and f-MgO contents in BOFS, including hydration treatment [18,19], heat treatment [20,21], accelerated carbonation [22,23], and natural weathering [24,25]. More recently, a hot-slag modification process has been introduced, in which oxygen and silica (SiO_2_) are injected into molten BOFS to oxidize metallic iron and facilitate the reaction of f-CaO with SiO_2_, thereby forming stable calcium silicate phases [26]. This process effectively eliminates expansion-inducing components and yields volume-stable modified BOFS (MBOFS). In addition, magnetic separation has been applied to remove metallic iron, further reducing expansion risks associated with iron oxidation [27]. Among these approaches, the hot-slag modification process has been regarded as the most effective technique, as it not only eliminates expansion-inducing components but also produces volume-stable aggregates suitable for concrete applications.

Traditional concrete is a brittle material with high compressive strength but low tensile and flexural strength, resulting in limited deformation capacity. To overcome this limitation, various fibers—such as basalt fiber, carbon fiber (CF), glass fiber, steel fiber, synthetic fiber, and natural fiber—have been incorporated into concrete to enhance its toughness and mechanical properties [28,29,30,31,32].

Among different fiber types, carbon fiber (CF) is distinguished by its unique properties, including low density, high strength, resistance to chemical corrosion and elevated temperatures, and excellent durability [33,34,35,36]. When incorporated into concrete, CF has been shown to enhance flexural, compressive, and tensile strength, while also improving impact resistance, fatigue performance, high-temperature stability, electrical conductivity, and chemical resistance [37,38,39,40,41]. Commercial CF filaments are generally coated with a silane-based sizing during the spinning process. This coating protects the fibers from mechanical damage and acts as a coupling agent in composites, thereby promoting strong interfacial bonding between CF and the surrounding matrix [42,43]. However, the sizing may hinder fiber dispersion, resulting in non-uniform distribution within the cement matrix or slippage during mechanical failure. Previous studies have demonstrated that fiber dispersion plays a critical role in determining the mechanical performance of concrete, with more uniform distribution leading to enhanced strength and overall behavior [44,45,46].

Heat treatment has been identified as the most effective method for removing the sizing from the surface of CF, with the optimal treatment temperature reported to range between 530 °C and 550 °C [47,48]. In addition, pneumatic dispersion can be applied prior to the incorporation of chopped CF to reduce agglomeration and promote a more uniform fiber distribution within the concrete matrix [49]. The present study aims to evaluate the quasi-static and dynamic mechanical behavior of carbon fiber reinforced concrete (CFRC) in which modified basic oxygen furnace slag (MBOFS) fully replaces natural aggregates. Chopped CF was pretreated by heat treatment and pneumatic dispersion, and the effects of pretreatment were characterized using thermogravimetric analysis (TGA) and single-fiber tensile tests. The mechanical performance of MBOFS CFRC was investigated under quasi-static and dynamic loading conditions, including compressive strength, flexural strength, splitting tensile strength, and impact resistance. The dynamic increase factor (DIF), obtained from the reverse split Hopkinson pressure bar (RSHPB) test, was further employed to analyze the high-strain-rate response of the material.

This study aims to investigate the quasi-static and dynamic mechanical behavior of CFRC in which MBOFS fully replaces natural aggregates. Chopped CF were pretreated by heat treatment and pneumatic dispersion, and their effects were characterized through TGA and single-fiber tensile tests. The mechanical performance was further evaluated under various loading conditions, including compressive, flexural, splitting tensile, impact, and RSHPB tests.

## 2. Materials

This section describes the materials employed in this study, including chopped carbon fiber (CF), modified basic oxygen furnace slag (MBOFS), natural aggregates, and cement, as well as the mix proportions used for preparing the concrete specimens.

### 2.1. Chopped Carbon Fiber

- Thermogravimetric analysis of CF

Chopped polyacrylonitrile-based carbon fibers (CF) with lengths of 6 mm and 12 mm (TC36P, Tairylan Division, Formosa Plastics Group, Taipei, Taiwan) were incorporated to reinforce the concrete. Prior to mixing, the surface sizing was removed through thermal pretreatment. To determine the optimal treatment temperature and duration, thermogravimetric analysis (TGA) was conducted using a STA7300 analyzer (Hitachi High-Tech Corp., Tokyo, Japan). The CF sample was placed in a ceramic crucible under a nitrogen flow of 100 mL/min, and two heating procedures were applied as follows:

1. Heating from 40 °C temperature to 700 °C at 20 °C/min.
2. Heating from 40 °C to 550 °C at 20 °C/min, holding for 30 min, then heating to 700 °C at 20 °C/min.

**Figure 1:**
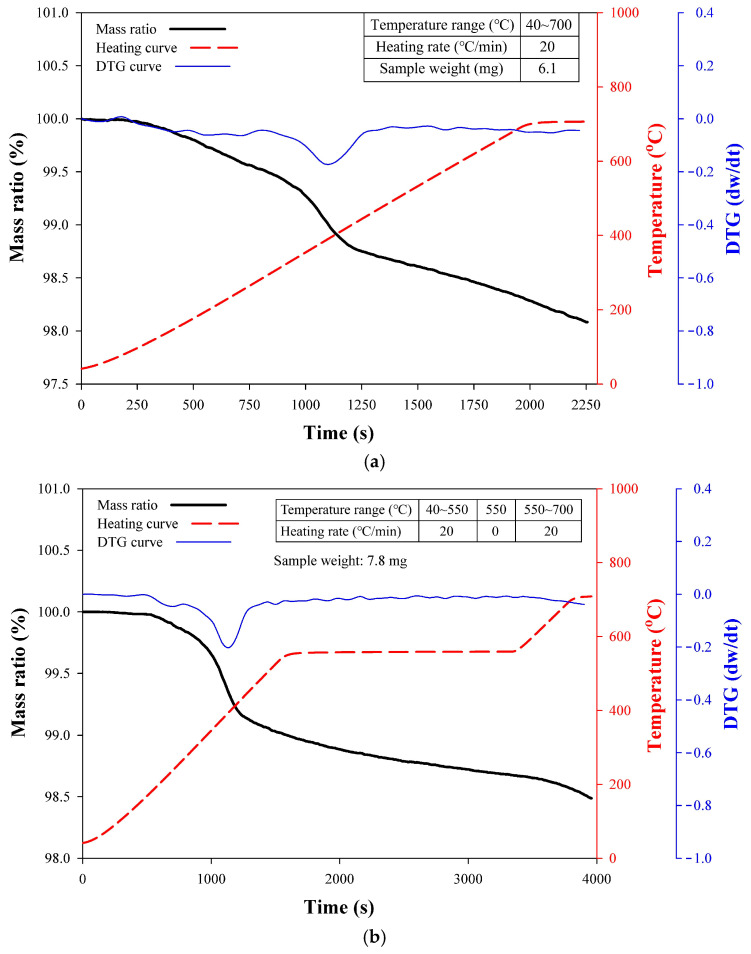
TGA results of the two heating procedures: (**a**) continuous heating up to 700 °C, and (**b**) holding at 550 °C for 30 min.

The corresponding TGA results are presented in Figure 1a,b. In the first procedure (Figure 1a), significant weight loss was observed near 400 °C based on the derivative thermogravimetric (DTG) result, indicating rapid burnout of the surface sizing. The mass ratio curve (black) exhibited a gradual weight reduction of approximately 2% across the full temperature range, with a pronounced drop occurring between 300 °C and 500 °C, which is likely associated with the degradation of sizing and partial organic constituents. The DTG curve (blue) confirmed this as the primary degradation stage, while the heating curve (red) reflected the controlled temperature increase. These results indicate the removal of sizing and provide insight into the thermal degradation behavior of the fibers below 700 °C.

In the second procedure (Figure 1b), where the sample was heated to 550 °C and held for 30 min, the total weight loss was approximately 1.6%. A sharp weight reduction between 300 °C and 500 °C was evident in both the TGA and DTG curves, again corresponding to the decomposition of surface sizing and organic residues. During the isothermal stage at 550 °C, the mass remained relatively stable, confirming that the major decomposition process was complete. Further heating to 700 °C resulted in only minor additional weight loss. These findings demonstrate that most volatile and organic constituents decompose below 550 °C, while the fibers exhibit good thermal stability beyond this temperature.

**Figure 2:**
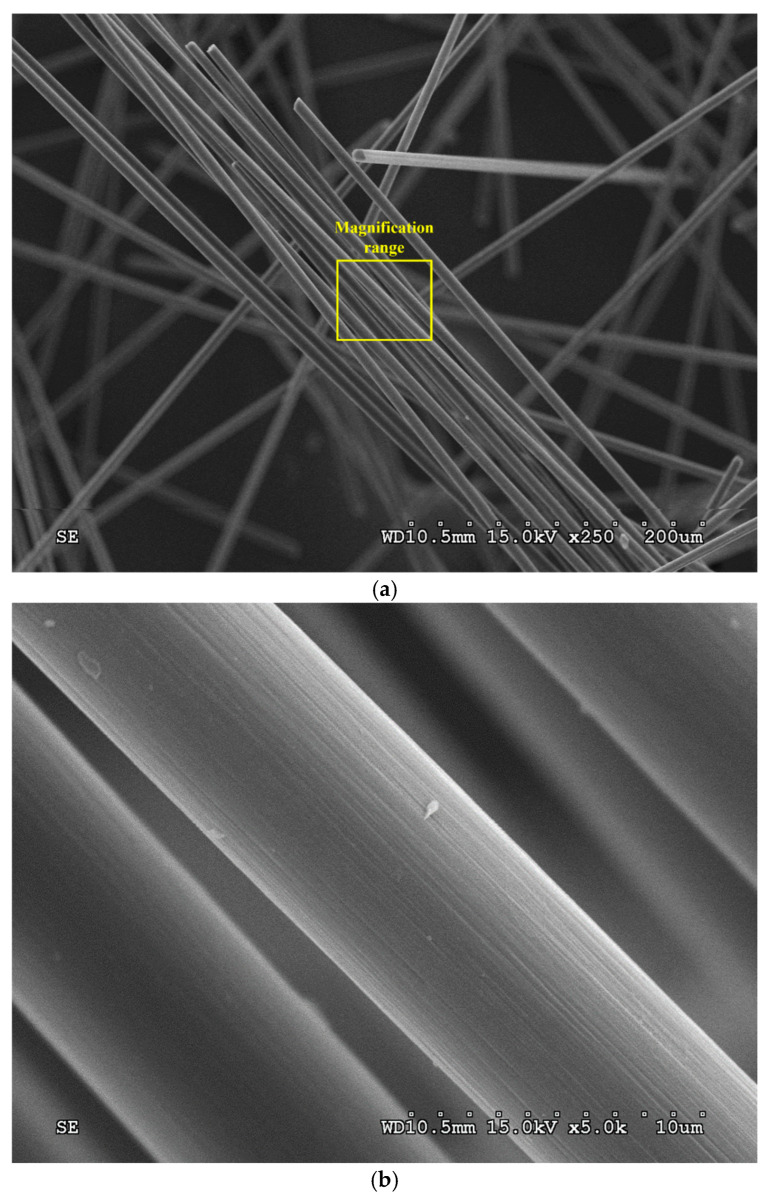
SEM micrographs of CF after TGA: (**a**) 250× magnification; (**b**) 5000× magnification.

Additionally, the surface morphology of CF specimens subjected to TGA was examined using a S3000H scanning electron microscope (SEM) (Hitachi High-Tech Corp., Tokyo, Japan) to verify the removal of surface sizing and assess structural integrity. The SEM micrographs at magnifications of 250× and 5000× are presented in Figure 2a,b, respectively. The results confirmed that most of the surface sizing was removed after thermal treatment, demonstrating that heat treatment is an effective method for eliminating sizing from CF.

TGA results indicated that the surface sizing of CF rapidly burned off at ~500 °C, with a total weight loss of ~1.5% after holding at 550 °C. SEM micrographs further confirmed that most of the sizing was removed, demonstrating the effectiveness of heat treatment.

- Tensile strength of CF filament

**Figure 3:**
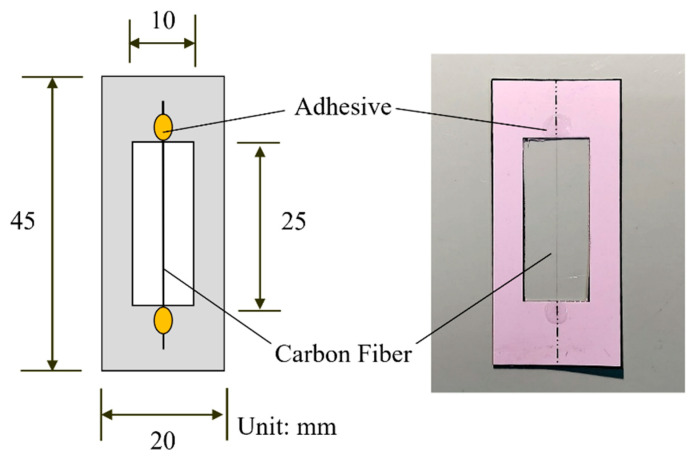
Schematic and photograph of the single-fiber tensile test specimen.

The tensile strength of CF filament, with and without heat treatment, was evaluated through single-fiber tensile tests conducted in accordance with ASTM C1557-20 [50]. Each fiber was straightened and mounted in a cardboard frame using adhesive to prevent slippage and bending. A schematic diagram and a photograph of the specimen are presented in Figure 3. The frame was clamped between the grips of a QC-528M1 material testing machine (Cometech, Taipei, Taiwan) to ensure alignment. After cutting both sides of the frame, the fiber was loaded at 0.5 mm/min until fracture. A total of 30 specimens were tested for each condition. For the single-fiber tensile tests, 30 individual fibers were tested for each condition (with and without heat treatment).

Figure 3 illustrates the single-fiber tensile test specimen, and the results are summarized in Table 1. Untreated CF exhibited an average tensile strength of 11.90 gf and an average displacement of 0.34 mm, while heat-treated CF showed 12.46 gf and 0.35 mm, respectively. Fiber stiffness was estimated from the slope of the force–displacement curve, and the calculated values were used for statistical analysis. Heat treatment slightly increased tensile strength but did not notably affect displacement.

Assuming normal distributions for both datasets, Levene’s test was performed at a 0.05 significance level with 30 samples per group using IBM SPSS Statistics Ver. 23. The resulting F-value of 0.766 with a p-value of 0.385 indicated homogeneity of variance. Accordingly, a t-test assuming equal variances was applied, yielding a t-value of −1.963 with a *p*-value of 0.0545. As this value exceeded the 0.05 threshold, the null hypothesis could not be rejected. Although the result was close to the significance level, indicating a possible trend, it did not reach statistical significance. Therefore, no significant differences were detected between untreated CF and heat-treated CF in terms of tensile strength, displacement, or stiffness. These findings suggest that heat treatment did not have a statistically significant effect on the single-fiber tensile properties of CF.

- Pneumatic dispersion of CF

**Figure 4:**
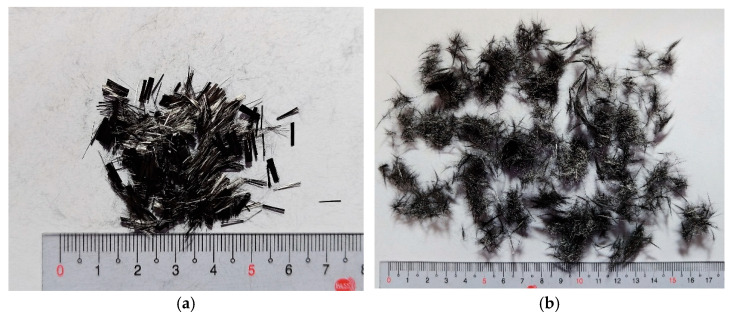

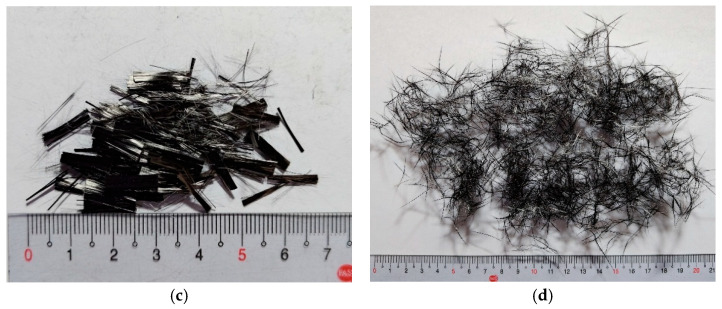
Photographs of heat-treated CF before and after pneumatic dispersion: (**a**) 6 mm CF before dispersion; (**b**) 6 mm CF after dispersion; (**c**) 12 mm CF before dispersion; (**d**) 12 mm CF after dispersion.

The pneumatic dispersion method consists of injecting high-pressure airflow into a sealed cylindrical container to separate chopped fiber bundles. In this process, CF is placed in the container, the lid is sealed, and compressed air is introduced, causing the fibers to collide and disperse. Prior to mixing, CF was pneumatically dispersed using a 3 hp air compressor to promote uniform distribution within the concrete. The appearance of 6 mm and 12 mm CF before and after pneumatic dispersion is shown in Figure 4. Appearance of 6 mm and 12 mm CF before and after pneumatic dispersion showing improved fiber separation.

### 2.2. Modified Basic Oxygen Furnace Slag and Natural Aggregate

**Figure 5:**
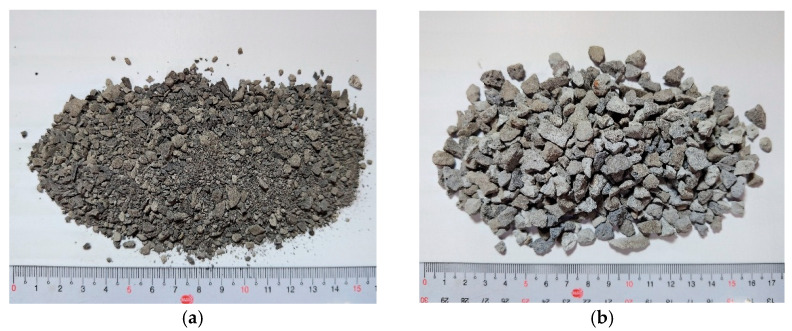
Appearance of MBOFS after mechanical crushing and sieving: (**a**) fine aggregate, (**b**) coarse aggregate.

**Figure 6:**
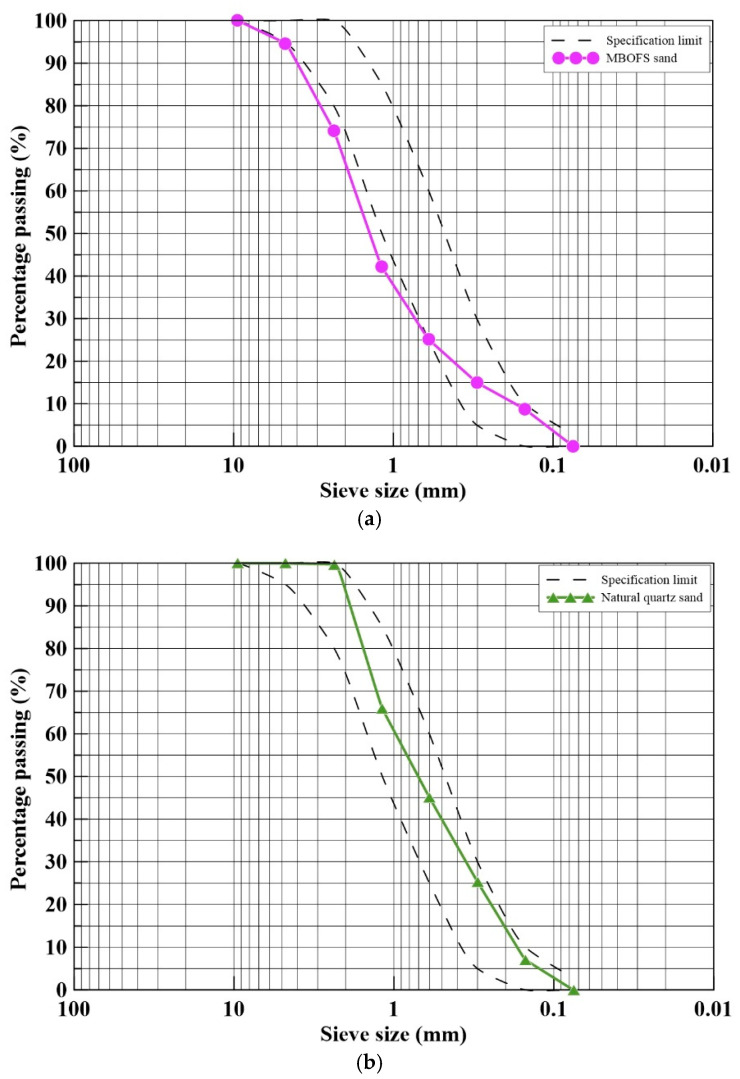
Particle size distribution curves of both aggregates: (**a**) MBOF aggregate and (**b**) natural aggregate.

The modified basic oxygen furnace slag (MBOFS) used in this study was supplied by China Steel Corporation (Kaohsiung, Taiwan). The material was mechanically crushed and sieved, and its appearance is shown in Figure 5. Sieve analyses of MBOFS fine aggregates and natural fine aggregates were carried out in accordance with ASTM C33/C33M-24a [51]. The fineness modulus (F.M.) of the MBOFS fine aggregate was 3.40, compared with 2.57 for the natural fine aggregate. The particle size distribution curves of both aggregates are shown in Figure 6a,b.

Specific gravity and water absorption tests of MBOFS and natural coarse aggregates were performed in accordance with ASTM C127-24 [52]. The MBOFS coarse aggregate exhibited a specific gravity of 3.23 and a water absorption of 1.54%, whereas the corresponding values for the natural coarse aggregate were 2.61 and 0.54%, respectively. The results are summarized in Table 2.

### 2.3. The Preparation of Modified Basic Oxygen Furnace Slag Carbon Fiber Reinforced Concrete

Type I Portland cement used in this study was supplied by Taiwan Cement Corporation (Taipei, Taiwan). Concrete specimens were prepared with a mix ratio of cement/fine aggregate/coarse aggregate = 1 : 1.65 : 2.475 (by weight), and a water-to-cement ratio of 0.55. Natural coarse and fine aggregates were fully replaced with MBOFS to produce MBOFS concrete specimens. All concrete specimens were cured for 28 days prior to the mechanical tests.

CF with lengths of 6 mm and 12 mm were incorporated into the concrete at 1% by weight of cement to produce fiber-reinforced concrete specimens. It has been reported that the optimal mechanical performance is achieved at a carbon fiber content of 1 wt.% [53]. Compressive and tensile strengths have been observed to decrease when the dosage deviates from this optimum. Furthermore, excessive CF content has been shown to adversely affect workability by increasing the mixture viscosity and hindering proper handling [54,55].

## 3. Test Methods

The mechanical properties of MBOFS CFRC were evaluated through a series of tests, including workability, static mechanical, and dynamic impact performance assessments. We prepared three replicate specimens for each condition in the compressive, flexural, and splitting tensile strength tests, as well as for the drop-weight impact and RSHPB tests.

### 3.1. Slump Test

Slump tests were performed on BOFS concrete with varying CF lengths in accordance with ASTM C143/C143M-20 [56] to evaluate workability. According to the ASTM standard, an acceptable slump range is between 15 mm and 230 mm.

### 3.2. Compressive Strength Test

Compressive strength tests were carried out using a universal testing machine in accordance with ASTM C39M-23 [57]. The cylindrical specimens measured ϕ100 mm × 200 mm, and the loading rate was set at 0.25 MPa/s.

### 3.3. Flexural Strength Test

Flexural strength tests were conducted using a universal testing machine (HT-9501, Hung-Ta Instrument Co., Ltd., Taipei, Taiwan); the tests were carried out using a four-point bending scheme in accordance with ASTM C78M-22 [58]. Specimens measured 280 mm × 70 mm × 70 mm (length × width × height). The loading rate was set at 0.98 MPa/min.

### 3.4. Splitting Tensile Strength Test

Splitting tensile strength tests were performed using a universal testing machine (HT-9501, Hung-Ta Instrument Co., Ltd., Taipei, Taiwan) in accordance with ASTM C496M-17 [59]. Cylindrical specimens with dimensions of ϕ100 mm × 200 mm were tested under a loading rate of 0.95 MPa/min.

### 3.5. Drop-Weight Test

**Figure 7:**
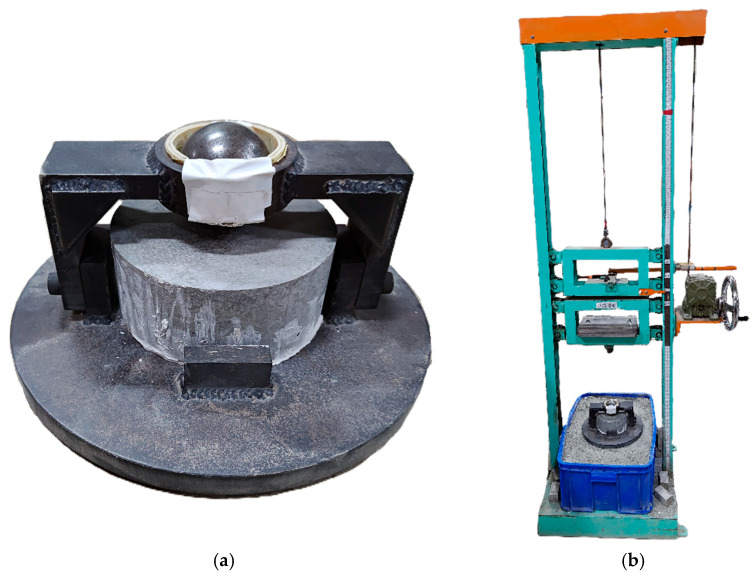
Drop-weight impact test setup: (**a**) specimen with the fixture, (**b**) drop-weight apparatus.

The drop-weight impact test was conducted using a drop-weight apparatus in accordance with ACI 544.2R-89 [60]. Cylindrical specimens with dimensions of ϕ152 mm × 63.5 mm were used. The testing setup, including the drop-weight device and the mounted specimen, is illustrated in Figure 7. Steel plates were attached to the impact head to increase the total impact mass, and the drop height was adjusted to obtain target impact energies of 75, 100, and 150 J. During the test, the impact head repeatedly struck the specimen until failure occurred. The number of impacts to failure was recorded and used to compare the impact resistance of different specimen types.

### 3.6. Stress Reversal Split Hopkinson Pressure Bar Test

**Figure 8:**
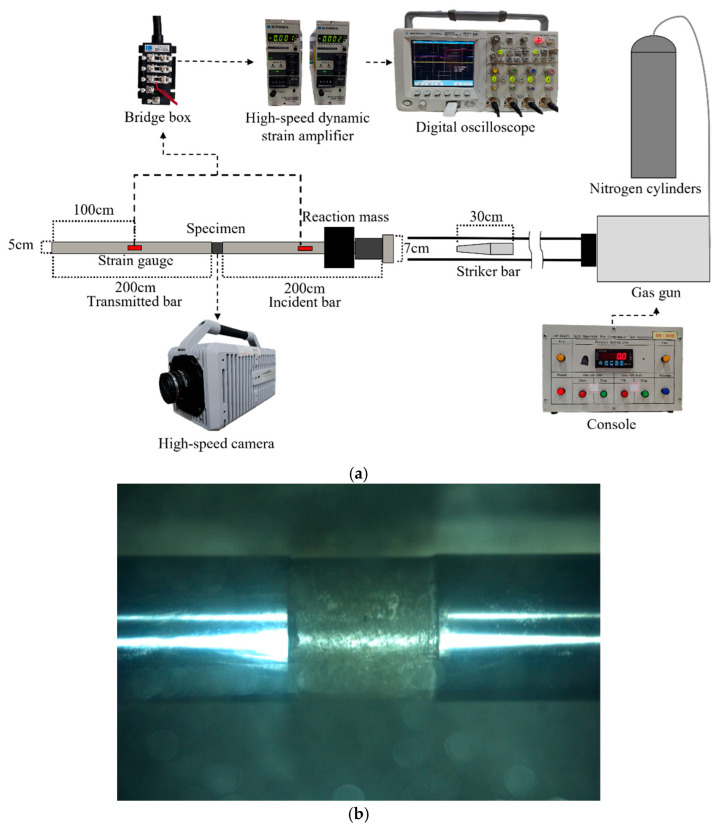
The stress reversal split Hopkinson pressure bar; (**a**) system setup and (**b**) concrete specimen between the incident and transmitted bars.

High-strain-rate dynamic tests were conducted using the stress reversal split Hopkinson pressure bar (RSHPB), as illustrated in Figure 8. Cylindrical specimens with dimensions of ϕ50 mm × 50 mm were tested. The stress reversal technique was applied to suppress multiple stress wave transmissions through the specimen. RSHPB permits only the first stress wave to pass through the specimen, ensuring that failure occurs under a single stress wave loading. Specifically, when the striker bar generates a compressive stress wave in the incident bar, a tensile stress wave is induced by the reaction mass to detach the incident bar from the specimen, thereby preventing repeated loading from reflected waves [61].

During the RSHPB test, strain gauges were mounted on both the incident and transmission bars to record the strain-time histories of the incident wave $\varepsilon_{i}(t)$, reflected wave $\varepsilon_{r}(t)$, and transmitted wave $\varepsilon_{t}(t)$. Based on these signals, the specimen’s average strain $\varepsilon_{avg}(t)$, average strain rate $\dot{\varepsilon }\left(t\right)$, and average stress $\sigma_{avg}\left(t\right)$ were calculated using the one-wave analysis method. The corresponding equations are presented in Equations (1)–(3):

(1) $$\varepsilon_{avg}\left(t\right)=-\frac{2C_{e}}{L_{s}}\int_{0}^{t}\left(\varepsilon_{r}\right)dt$$

(2) $$\dot{\varepsilon }\left(t\right)=-\frac{2C_{e}}{L_{s}}\varepsilon_{r}$$

(3) $$\sigma_{avg}\left(t\right)=\frac{E_{e}\cdot A_{e}}{A_{s}}\cdot \varepsilon_{t}\left(t\right)$$

where $C_{e}$ is the wave speed; E is the elastic modulus of the incident bar; $A_{e}$ is the cross-sectional area of incident bar; $L_{s}$ and $A_{S}$ are the length and cross-sectional area of the specimen, respectively.

## 4. Result and Discussion

This section presents the experimental results of MBOFS CFRC under quasi-static and dynamic loading. The mechanical performance was evaluated through slump tests, compressive strength, flexural strength, and splitting tensile strength tests, as well as impact resistance assessment. In addition, the dynamic increase factor (DIF) was determined using the RSHPB test.

The specimen-naming convention was defined as follows. The first letter denotes the type of mechanical test: slump (SL), compressive (C), flexural (F), splitting tensile (S), impact (I), and RSHPB (R). The second part specifies the aggregate type, where “B” represents the benchmark with natural aggregate and “MS” indicates modified BOFS concrete. The final part refers to the length of chopped CF, such as “CF12” for 12 mm fibers. For example, C-MS-CF6 designates a compressive test specimen prepared with MBOFS concrete and 6 mm CF.

### 4.1. Slump Test Results

The workability of MBOFS concrete incorporating chopped CF was evaluated through slump tests, with CF lengths of 6 mm and 12 mm added at 1.0% by weight of cement. The results are summarized in Table 3. As shown in Table 3, the slump values of MBOFS concrete were lower than those of the benchmark mixture with natural aggregates. This reduction is attributed to the angular particle shape of MBOFS aggregates (Figure 4), which increases internal friction and reduces flowability, as well as their slightly higher water absorption (Table 2), which decreases the amount of free water available for lubrication. Furthermore, the inclusion of chopped CF increased the viscosity of the fresh mixture due to fiber–matrix interactions and the tendency of fibers to interlock, further hindering flow. To mitigate these drawbacks, possible measures include optimizing fiber content and aspect ratio and employing suitable superplasticizers to enhance workability and more accurately characterize the flow behavior of fiber-reinforced mixtures.

### 4.2. Compressive Strength Test Results

Table 4 presents the average 28-day compressive strength of all specimens. The benchmark specimen C-B exhibited an average compressive strength of 30.30 MPa. Compared with C-B, specimen C-MS demonstrated a 24.75% increase, while C-MS-CF6 and C-MS-CF12 showed further improvements of 45.89% and 41.29%, respectively. Relative to C-MS, the addition of 6 mm and 12 mm chopped CF improved compressive strength by 16.95% and 9.23%, respectively. These results confirm that replacing natural aggregates with MBOFS substantially enhances compressive strength, primarily due to the higher density, angular shape, and rougher surface texture of MBOFS aggregates. Moreover, the incorporation of chopped CF provides additional reinforcement by bridging microcracks and restraining crack propagation under load. Between the two fiber lengths, 6 mm CF performed better than 12 mm CF, likely because shorter fibers disperse more uniformly and develop more efficient stress transfer within the matrix.

### 4.3. Flexural Strength Test Results

Table 5 summarizes the average 28-day flexural strength of all specimens. The benchmark specimen F-B exhibited a flexural strength of 4.37 MPa. Compared with F-B, the replacement of natural aggregates with MBOFS (F-MS) resulted in a 14.41% increase, while the addition of chopped CF led to substantial further improvements, with F-MS-CF6 and F-MS-CF12 showing increases of 45.29% and 54.34%, respectively. Relative to F-MS, the incorporation of 6 mm and 12 mm CF enhanced flexural strength by 26.99% and 34.91%. These results indicate that both MBOFS aggregate substitution and fiber reinforcement contribute positively to flexural strength, although the effect of fibers is more pronounced. The improvement is attributed to the ability of chopped CF to bridge cracks and restrain their propagation under bending, thereby increasing ductility and load-bearing capacity. Moreover, the superior performance of 12 mm CF compared with 6 mm CF suggests that longer fibers are more effective in flexural applications, as they provide greater anchorage within the cement matrix and better crack-bridging efficiency. From a structural perspective, the combined use of MBOFS and appropriately selected CF

### 4.4. Splitting Tensile Strength Test Results

Table 6 presents the average 28-day splitting tensile strength of all specimens. The benchmark specimen S-B exhibited a splitting tensile strength of 2.35 MPa. Compared with S-B, S-MS showed a 9.1% increase, while S-MS-CF6 and S-MS-CF12 achieved much greater improvements of 41.08% and 42.23%, respectively. Relative to S-MS, the addition of 6 mm and 12 mm chopped CF enhanced splitting tensile strength by 29.31% and 30.36%, respectively. These results confirm that both the replacement of natural aggregates with MBOFS and the incorporation of chopped CF contribute positively to splitting tensile strength, with fibers exerting a more significant reinforcing effect. The improvement is attributed to the ability of fibers to arrest and bridge cracks under tensile stresses, thereby delaying crack initiation and propagation. Although both fiber lengths demonstrated comparable strengthening effects, the slightly better performance of 12 mm CF suggests that longer fibers provide improved anchorage and crack-bridging efficiency under tensile loading.

### 4.5. Drop-Weight Test Results

Drop-weight impact tests were carried out at three energy levels: 75 J, 100 J, and 150 J. Table 7 summarizes the applied impact energy and the corresponding number of impacts to failure for all specimens. As shown in Table 7, specimens subjected to the lowest energy level (75 J) required a greater number of impacts to reach failure, whereas those tested at the highest energy level (150 J) failed after fewer impacts, reflecting the higher severity of loading. At the same impact energy, MBOFS CFRC specimens consistently required more impacts to fail than their counterparts without CF, demonstrating the beneficial role of fibers in enhancing impact resistance and energy absorption capacity. Among the fiber-reinforced specimens, those with 12 mm CF sustained slightly more impacts before failure compared with those containing 6 mm CF. This indicates that longer fibers may contribute to more effective crack-bridging and energy dissipation mechanisms during repeated impacts.

**Figure 9:**
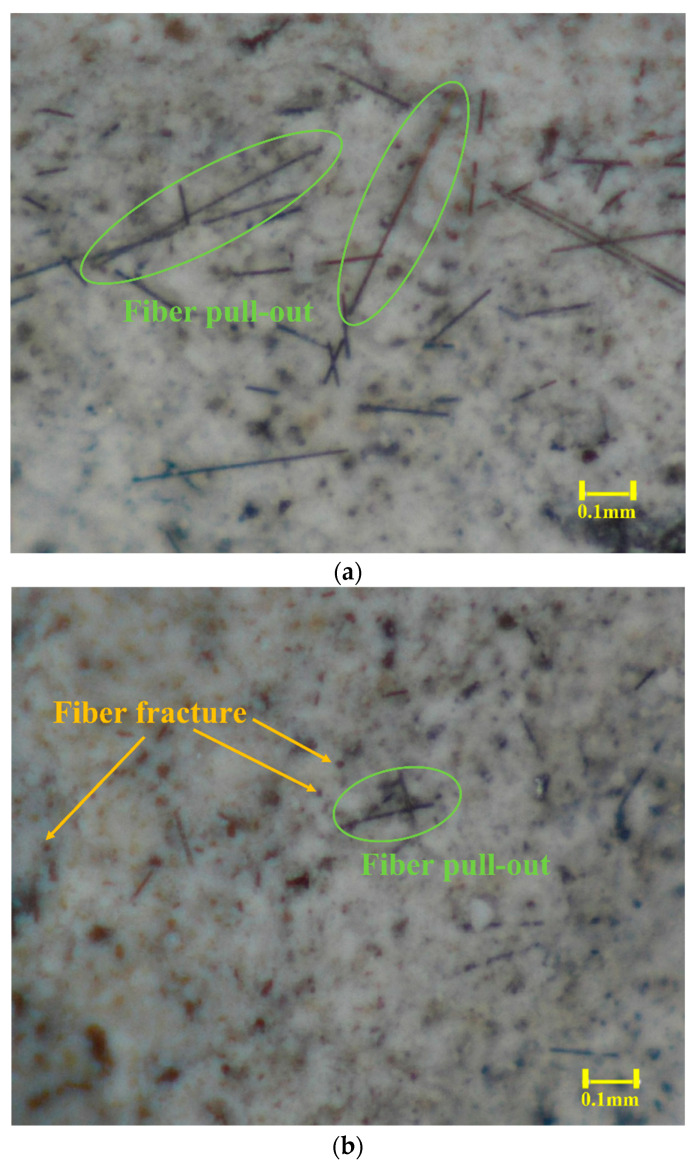
Optical microscope images of the specimens after drop-weight impact tests: (**a**) I-MS-CF6; (**b**) I-MS-CF12.

In addition, the fracture surfaces of the specimens damaged under drop-weight impact were examined using an optical microscope (Eakins-36MP, OPTO-EDU Co., Ltd., Beijing, China). The corresponding images of specimens I-MS-CF6 and I-MS-CF12 are presented in Figure 9a,b, respectively. As shown in Figure 9a, the predominant failure mode of I-MS-CF6 was fiber pull-out, whereas Figure 9b indicates that I-MS-CF12 primarily failed through fiber fracture.

### 4.6. RSHPB Test

**Figure 10:**
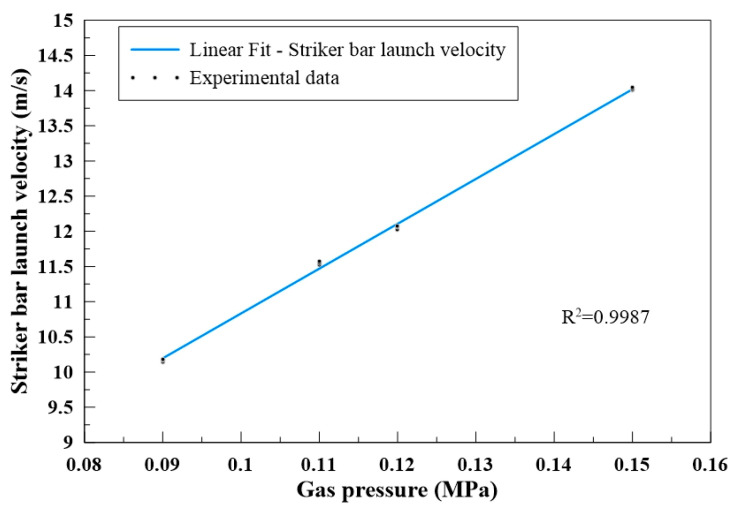
Relationship between gas pressure and striker bar launch velocity.

In this study, RSHPB tests were performed on MBOFS CFRC specimens R-MS-CF6 and R-MS-CF12. Four gas pressures (0.09, 0.11, 0.12, and 0.15 MPa) were applied to launch the striker bar, and the corresponding launch velocities were measured. The relationship between gas pressure and striker bar velocity is presented in Figure 10. As shown, the launch velocities were highly consistent for repeated trials at the same gas pressure, indicating stable loading conditions. Furthermore, a clear linear correlation was observed between gas pressure and launch velocity, which enables accurate control of impact loading conditions during testing. For data interpretation, the fracture point of each specimen was defined as 80% of the maximum stress, while the failure strain rate was defined as the strain rate corresponding to the maximum stress. This approach ensured consistency in evaluating the strain energy of all specimens tested under RSHPB loading.

**Figure 11:**
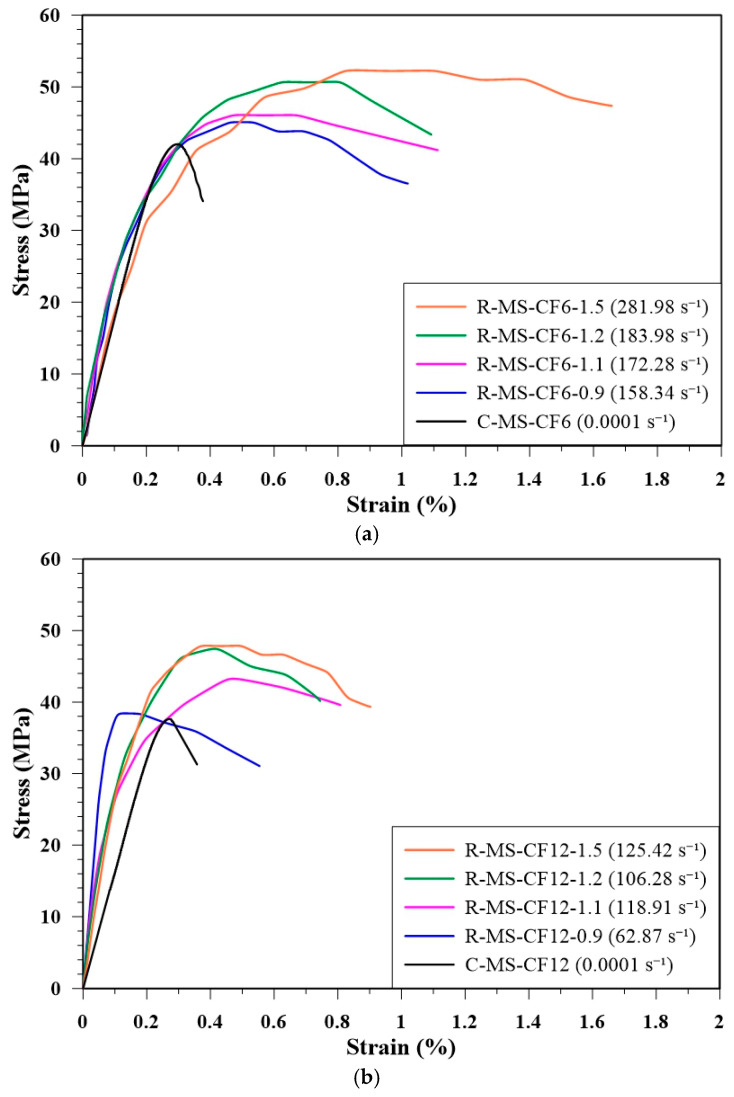
The stress–strain curves of (**a**) R-MS-CF6 and (**b**) R-MS-CF12 under different gas pressures.

Figure 11 shows the stress–strain curves of R-MS-CF6 and R-MS-CF12 at different gas pressures, together with quasi-static compressive curves for comparison. The dynamic curves demonstrate both increased peak stress and steeper slopes relative to quasi-static loading, highlighting the enhanced stiffness and strength of CFRC under high-strain-rate loading.

Table 8 summarizes the dynamic strength and corresponding failure strain rates of MBOFS CFRC specimens reinforced with 6 mm and 12 mm CF under different gas pressures (or striker bar launch velocities). The results indicate that higher striker bar velocities produced greater stress in the specimens, confirming the strain-rate sensitivity of MBOFS CFRC. Within the tested pressure range, R-MS-CF6 consistently exhibited higher dynamic strength than R-MS-CF12, suggesting that shorter fibers provide more effective stress transfer under high-strain-rate conditions.

**Figure 12:**
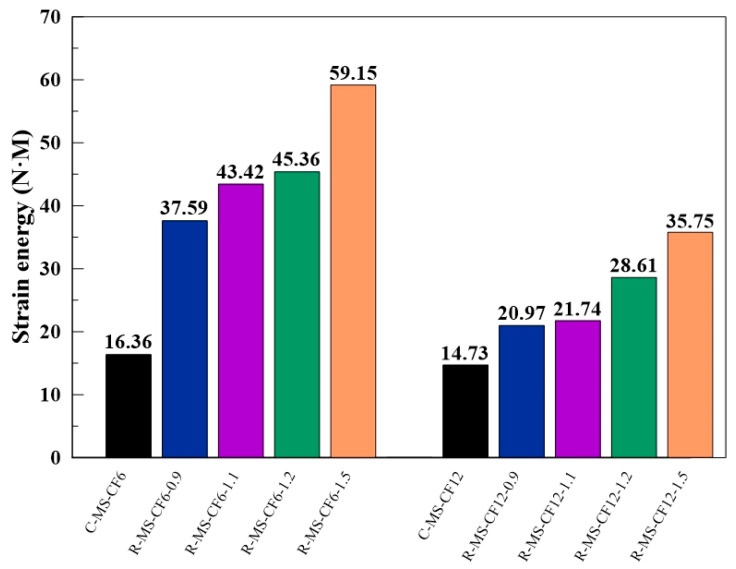
Strain energy of MBOFS CFRC specimens under RSHPB test.

The strain energy was calculated by integrating the area under the dynamic stress–strain curve and multiplying by specimen volume, representing the energy absorbed through deformation. Figure 12 presents the strain energy of all specimens under SRSHPB. The results reveal that strain energy increased with gas pressure (striker bar launch velocity), reflecting the greater energy absorption capacity of specimens at higher strain-rate loading. Moreover, at the same gas pressure, specimen R-MS-CF6 consistently absorbed more energy than specimen R-MS-CF12, reinforcing the conclusion that 6 mm CF provided superior dynamic strength and strain energy compared with 12 mm CF under dynamic impact.

**Figure 13:**
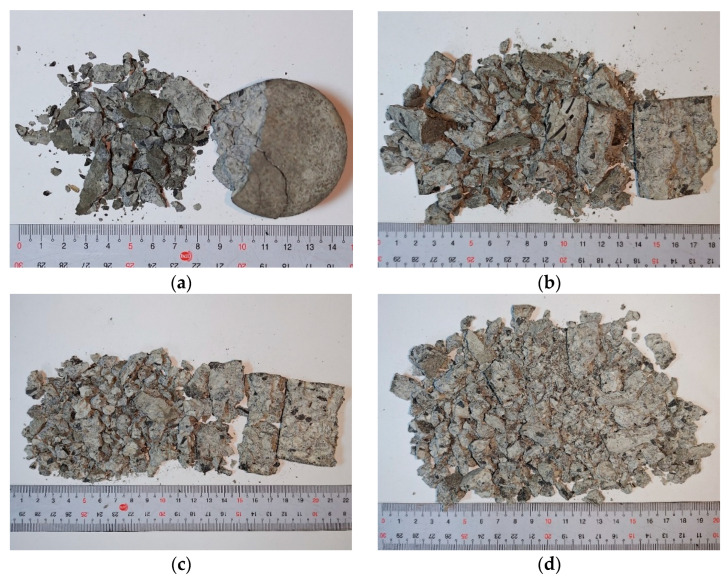
Dynamic failure photographs of R-MS-CF6 specimens under different gas pressures: (**a**) 0.09 MPa; (**b**) 0.11 MPa; (**c**) 0.12 MPa; (**d**) 0.15 MPa.

**Figure 14:**
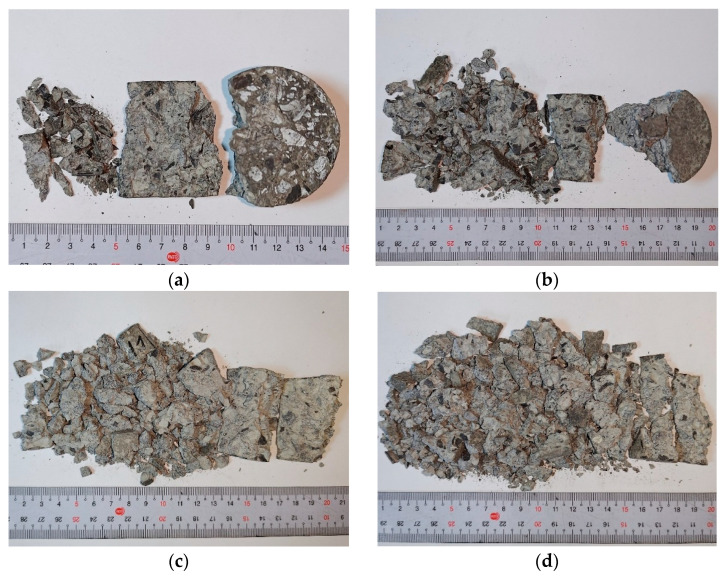
Dynamic failure photographs of R-MS-CF12 specimens under different gas pressures: (**a**) 0.09 MPa; (**b**) 0.11 MPa; (**c**) 0.12 MPa; (**d**) 0.15 MPa.

Failure photographs of specimens R-MS-CF6 and R-MS-CF12 under various gas pressures in the RSHPB tests are presented in Figure 13 and Figure 14, respectively. The images show that at lower gas pressures, the specimens remained relatively intact, while at higher gas pressures, the specimens exhibited severe fragmentation. The failure patterns of R-MS-CF6 and R-MS-CF12 were generally similar under the same dynamic loading conditions, indicating that fiber length had only a minor influence on the macroscopic failure mode, as illustrated in Figure 13 and Figure 14.

### 4.7. Dynamic Increase Factor

The dynamic increase factor (DIF) was employed to evaluate the extent of strength enhancement in MBOFS CFRC under dynamic loading. DIF_1_ is defined as the ratio of the dynamic compressive strength of MBOFS CFRC obtained from the RSHPB test to its corresponding quasi-static compressive strength, as expressed in Equation (4). DIF_2_ is defined as the ratio of the dynamic compressive strength of MBOFS CFRC measured in the RSHPB test to the quasi-static compressive strength of natural aggregate concrete (C-B), as shown in Equation (5).

(4) $${DIF}_{1}={f\prime }_{dynamic}/{f\prime }_{c}$$

(5) $${DIF}_{2}={f\prime }_{dynamic}/{f\prime }_{cB}$$

where ${f\prime }_{dynamic}$ is the dynamic compressive strength; ${f\prime }_{c}$ is the quasi-static compressive strength of MBOFS CFRC; ${f\prime }_{cB}$ is the quasi-static compressive strength of natural aggregate concrete.

**Figure 15:**
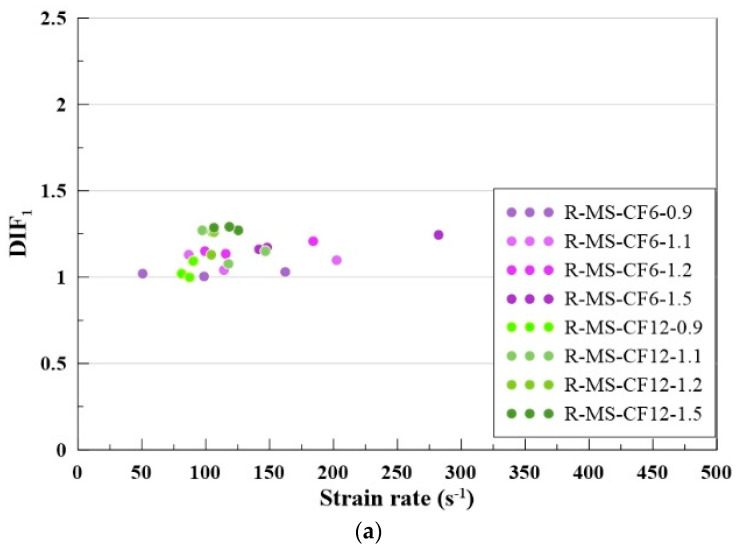

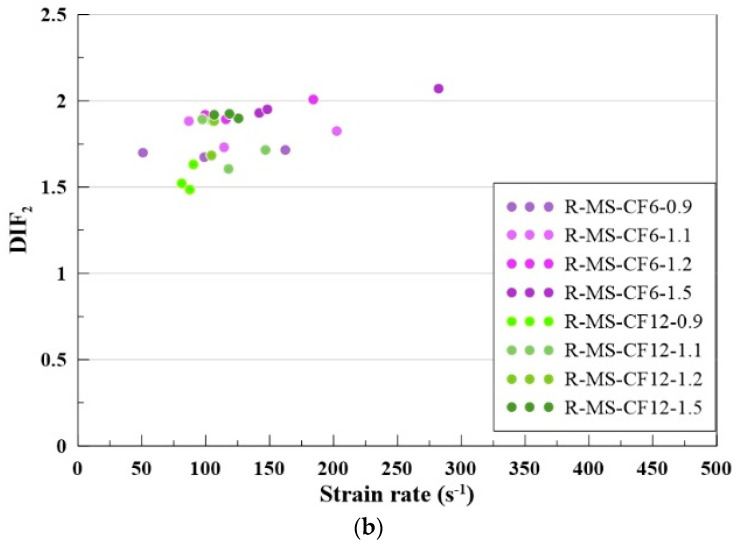
Scatter plots of dynamic increase factors (DIFs) versus strain rate: (**a**) DIF_1_; (**b**) DIF_2_.

The scatter plots of DIF_1_ and DIF_2_ are presented in Figure 15a,b, respectively. As shown in both figures, the values of DIF_1_ and DIF_2_ are greater than 1, indicating that the dynamic compressive strength of MBOFS CFRC specimens in the RSHPB tests exceeded their quasi-static compressive strength. A comparison of Figure 15a,b further reveals that the dynamic compressive strength of MBOFS CFRC specimens can reach up to twice the compressive strength of natural aggregate concrete (C-B) under quasi-static conditions.

In summary, MBOFS CFRC exhibited excellent strain-rate sensitivity, impact resistance, and energy absorption capacity under dynamic loading. The optimum fiber length depends on the loading condition. In the drop-weight impact test, specimens with 12 mm CF (I-MS-CF12) outperformed those with 6 mm CF (I-MS-CF6), as the dominant failure mechanism resembled splitting and flexural behavior. This observation is consistent with the results of quasi-static splitting tensile and flexural strength tests. In contrast, under high strain rate loading in the RSHPB test, specimens with 6 mm CF (R-MS-CF6) exhibited superior performance compared with those with 12 mm CF (R-MS-CF12), since the governing mechanical behavior was more closely related to compressive loading. This finding aligns with the results of quasi-static compressive strength tests. These outcomes highlight the importance of optimizing fiber geometry to tailor the dynamic response of MBOFS CFRC for different structural applications.

## 5. Conclusions

The following is a summary of the research findings on the physical properties of modified basic oxygen furnace slag (MBOFS) and carbon fiber (CF). Based on the results of quasi-static and dynamic mechanical performance tests conducted on CF-reinforced MBOFS concrete specimens, the following conclusions can be drawn:

1. Thermogravimetric analysis (TGA) revealed that heat-treated CF exhibited up to 1.5% weight loss due to the removal of surface sizing. Scanning electron microscopy (SEM) further confirmed the effective elimination of sizing through thermal treatment.
2. Single-fiber tensile tests indicated that heat treatment did not significantly influence the tensile strength of individual CF filaments.
3. Replacing natural aggregates with MBOFS reduced the slump of concrete mixtures but significantly enhanced compressive, flexural, and splitting tensile strengths.
4. The incorporation of chopped CF at 1% by weight of cement further improved the mechanical performance of MBOFS concrete. In quasi-static tests, specimens with 6 mm CF exhibited higher compressive strength, whereas those with 12 mm CF showed superior flexural and splitting tensile strengths.
5. Drop-weight impact tests demonstrated that MBOFS CFRC specimens exhibited greater impact resistance than non-fiber specimens. Among fiber-reinforced specimens, those with 12 mm CF showed slightly higher impact resistance than those with 6 mm CF.
6. RSHPB tests indicated that MBOFS CFRC specimens with 6 mm CF achieved higher dynamic strength than those with 12 mm CF. Increasing gas pressure led to higher dynamic strength, and the dynamic increase factor (DIF) showed an upward trend with increasing strain rate.
7. The results of this study indicate that MBOFS CFRC has the potential to utilize industrial by-products as sustainable construction materials.
8. The MBOFS material used in this work was supplied by China Steel Corporation (Kaohsiung, Taiwan), and its current production is limited to research purposes, which may constrain broader applicability at present. Future research will therefore focus on conducting large-scale field experiments and evaluating the long-term durability of CFRC incorporating MBOFS to further validate its practical potential.

## Figures and Tables

**Table 1:** Single-fiber tensile test results of CF.

No.	Untreated CF			Heat-Treated CF		
	Force (gf)	Displ. (mm)	Slope (gf/mm)	Force (gf)	Displ. (mm)	Slope (gf/mm)
1	8.96	0.27	33.82	10.84	0.28	38.86
2	12.99	0.39	33.57	12.85	0.34	37.35
3	12.29	0.36	33.75	13.30	0.36	36.73
4	13.33	0.37	36.32	12.01	0.35	34.82
5	9.99	0.30	33.19	14.57	0.40	36.69
6	14.36	0.40	35.54	12.72	0.36	35.53
7	9.80	0.28	35.24	11.07	0.33	33.94
8	9.04	0.27	33.37	13.09	0.38	34.72
9	12.50	0.33	37.52	13.09	0.37	35.38
10	10.84	0.32	34.20	9.56	0.27	35.92
11	10.76	0.33	32.91	10.04	0.26	39.06
12	11.00	0.32	34.92	14.70	0.41	36.02
13	12.53	0.36	34.89	12.16	0.35	35.24
14	14.29	0.42	34.11	14.82	0.40	36.78
15	12.29	0.34	35.72	11.32	0.33	34.62
16	14.78	0.41	36.12	11.69	0.34	34.29
17	10.79	0.31	35.38	12.32	0.31	39.23
18	11.48	0.33	34.90	11.84	0.33	35.54
19	11.80	0.33	35.66	10.74	0.33	32.76
20	12.53	0.37	33.67	11.98	0.37	32.73
21	10.84	0.32	34.31	10.25	0.28	37.26
22	13.59	0.38	35.85	11.16	0.30	36.96
23	13.68	0.38	36.49	13.38	0.36	36.85
24	11.64	0.31	37.93	10.15	0.29	34.76
25	11.19	0.30	36.94	12.53	0.34	36.95
26	11.72	0.35	33.98	15.47	0.43	36.39
27	14.23	0.42	34.20	15.42	0.43	36.02
28	13.72	0.38	35.72	12.53	0.40	31.47
29	10.52	0.31	33.50	12.19	0.34	35.44
30	9.41	0.26	36.62	15.90	0.44	35.97
**Avg.**	11.90	0.34	-	12.46	0.35	
**σ**	1.65	0.05	-	1.70	0.05	
**F value**	-	-	-	-	-	0.766
***p* value**	-	-	-	-	-	0.385
**t value**	-	-	-	-	-	−1.963
***p* value**	-	-	-	-	-	0.0545

**Table 2:** Specific gravity and absorbed moisture.

Specific Gravity	BOF	Natural
Specimen weight (g)	2000	2000
Oven-dry weight (g)	1992	1995.2
Saturated-surface-dry weigh (g)	2022.7	2006
Specimen weight in water (g)	1405	1241.7
Bulk specific gravity	3.23	2.61
Apparent specific gravity	3.39	2.65
Absorbed moisture (%)	1.54	0.54
Moisture content (%)	0.40	0.24

**Table 3:** Slump of the benchmark and MBOFS concretes.

Specimen	SL-B	SL-MS	SL-MS-CF6	SL-MS-CF12
Slump (mm)	239	130	48	36

**Table 4:** Compressive strength of the benchmark and MBOFS concretes.

Specimen	Compressive Strength (MPa)	Average Compressive Strength (MPa)	Increment Based on C-B (%)	Increment Based on C-MS (%)
C-B	29.88	30.30	-	-
	30.42			
	30.60			
C-MS	36.80	37.80	24.75	-
	38.24			
	38.37			
C-MS-CF6	42.95	44.21	45.89	16.95
	44.45			
	45.22			
C-MS-CF12	40.52	41.29	36.26	9.23
	41.67			
	41.67			

**Table 5:** Flexural strength of the benchmark and MBOFS concretes.

Specimen	Flexural Strength (MPa)	Average Flexural Strength (MPa)	Increment Based on F-B (%)	Increment Based on F-MS (%)
F-B	4.19	4.37	-	-
	4.22			
	4.69			
F-MS	4.59	5.00	14.41	-
	4.98			
	5.43			
F-MS-CF6	5.78	6.35	45.29	26.99
	6.55			
	6.71			
F-MS-CF12	6.42	6.74	54.34	34.91
	6.84			
	6.98			

**Table 6:** Splitting tensile strength of the benchmark and MBOFS concretes.

Specimen	Splitting Strength (MPa)	Average Splitting Strength (MPa)	Increment Based on S-B (%)	Increment Based on S-MS (%)
S-B	2.31	2.35	-	-
	2.37			
	2.38			
S-MS	2.17	2.57	9.1	-
	2.64			
	2.89			
S-MS-CF6	2.95	3.32	41.08	29.31
	3.42			
	3.60			
S-MS-CF12	2.85	3.35	42.23	30.36
	3.22			
	3.98			

**Table 7:** Impact energy and number of impacts for the benchmark and MBOFS concretes.

Specimens	Impact Energy (J)	Impact Number			Average Impact Number
I-B	150	2	3	5	3.3
	100	8	9	9	8.7
	75	27	28	34	29.7
I-MS	150	4	4	4	4
	100	8	9	16	11
	75	65	133	185	127.7
I-MS-CF6	150	9	10	11	10
	100	14	16	30	20
	75	280	376	388	348
I-MS-CF12	150	9	10	12	10.3
	100	19	20	29	22.7
	75	401	457	489	449

**Table 8:** Failure strain rate and strength of MBOFS specimens with 6 mm and 12 mm fibers under various gas pressures.

Specimens	Gas Pressure (MPa)	Failure Strain Rate (s^−1^)	Strength (MPa)
R-MS-CF6	0.09	158.34	45.03
	0.11	172.28	46.04
	0.12	183.98	50.64
	0.15	281.98	52.22
R-MS-CF12	0.09	62.87	38.34
	0.11	118.91	43.22
	0.12	106.28	47.45
	0.15	125.42	47.84

## Data Availability

The original contributions presented in this study are included in the article. Further inquiries can be directed to the corresponding author.
